# Maternal diet and gestational diabetes mellitus modestly influence children's growth during their first 24 months

**DOI:** 10.1002/jpn3.70098

**Published:** 2025-06-09

**Authors:** Lotta Saros, Tero Vahlberg, Ella Koivuniemi, Noora Houttu, Kristiina Tertti, Nitin Shivappa, James R. Hébert, Harri Niinikoski, Kirsi Laitinen

**Affiliations:** ^1^ Integrative Physiology and Pharmacology Unit, Institute of Biomedicine University of Turku Turku Finland; ^2^ Department of Biostatistics University of Turku and Turku University Hospital Turku Finland; ^3^ Nutrition and Food Research Center University of Turku Turku Finland; ^4^ Department of Obstetrics and Gynaecology University of Turku and Turku University Hospital Turku Finland; ^5^ Cancer Prevention and Control Program and Department of Epidemiology and Biostatistics, Arnold School of Public Health University of South Carolina Columbia South Carolina USA; ^6^ Department of Nutrition Connecting Health Innovations LLC Columbia South Carolina USA; ^7^ Department of Pediatrics and Adolescent Medicine Turku University Hospital and University of Turku Turku Finland

**Keywords:** adiposity, head circumference, height, maternal diabetes, nutrition, offspring

## Abstract

**Objective:**

To evaluate whether diet and gestational diabetes mellitus (GDM) during pregnancy influence children's growth during their first 24 months.

**Methods:**

Growth data of children (*n* = 378) of women with overweight/obesity were obtained from clinic records (birth, 3, 6, 12 and 24 months), and variables (standard deviation scores (SDS) or percentages) were calculated based on Finnish growth charts. Body composition was measured by air displacement plethysmography (*n* = 73, 24 months). Diet was assessed (diet quality index, nutrient intakes and diet inflammatory index (DII®)) in early and late pregnancy. GDM was determined by an oral glucose tolerance test.

**Results:**

A good dietary quality in early pregnancy associated positively with the children's height at each time point (adj. mean difference range = 0.28–0.30 SDS, *p* < 0.05) and head circumference at 12 and 24 months (adj. mean difference range = 0.38–0.42 SDS, *p *< 0.05). A good dietary quality in late pregnancy associated with a lower fat mass in children (adj. mean difference = −0.69, *p* < 0.05). A higher DII was correlated with a higher weight at 24 months but a reduced height at each time point (adj. *p* < 0.05). GDM associated negatively with the children's head circumference at birth and 6 months (adj. mean difference range = −0.43 to [−0.22] SDS, *p* < 0.05).

**Conclusions:**

Consuming a good quality diet during pregnancy associated with a greater infantile height and head circumference but a lower adiposity in 2‐year‐old children. GDM may lead to a slightly smaller head circumference in early infancy. Mothers with overweight or obesity could support their children's growth by consuming a good quality diet, with low inflammatory potential during pregnancy.

**Trial Registration:**

ClinicalTrials.gov Identifier: NCT01922791, 14 August 2013.

## Abstract (highlights)

**What is Known:**

- Children of mothers with overweight and/or gestational diabetes mellitus (GDM) have an increased risk of overweight.
- Preliminary evidence suggests that a healthy diet during pregnancy has beneficial effects on a child's growth, especially lower overweight risk.

**What is New:**

- An overall good‐quality diet, including foods with lower inflammatory potential, in early pregnancy associated with higher height and head circumference standard deviation scores but lower adiposity in infants born to mothers with overweight/obesity.
- GDM in mothers with overweight or obesity may lead to a smaller child's head circumference in early infancy.

## 1 INTRODUCTION

Considering the global overweight and obesity burden (body mass index [BMI] ≥ 25 and BMI ≥ 30 kg/m^2^),^1^ an increasing number of children are being exposed already in utero to this condition and its comorbidities, such as gestational diabetes mellitus (GDM).^2^ Indeed, these conditions are linked to higher weight from birth to 4 years^3^, ^4^ and risk for overweight/obesity in 12‐year‐old childen.^4^, ^5^, ^6^ Obesity and GDM may trigger changes in maternal metabolism, leading to hyperglycaemia, and altered hormone levels (e.g., adipokines and leptin), as well as increased levels of low‐grade inflammation and epigenetic changes.^7^, ^8^ Modification of early‐life circumstances, particularly diet, might provide an opportunity to diminish these unfavourable effects and to support optimal growth.

Previous research has shown that a good dietary quality (i.e., consumption of vegetables, fruits, whole‐grains and fish) during pregnancy could lower children's weight and body fat percentage at birth and 6 months.^9^ In addition, a health‐promoting diet quality has been linked with higher weight and height at birth,^10^ although another report did not detect these associations.^11^ Individual nutrients may also influence children's growth. A higher intake of *n* − 3 polyunsaturated fatty acids (*n* − 3 PUFAs) during pregnancy associated with lower weight and fat mass at birth and lower obesity risk in 3‐year‐old children.^12^, ^13^ In contrast, higher maternal consumption of total fat and saturated fatty acids (SFAs) have been linked with a higher body fat percentage in 6‐month‐old children.^14^ The potential mechanism linking maternal diet and children's growth may be the presence of diet‐induced inflammation due to higher consumption of SFA, while *n* − 3 PUFA has opposite effects.^15^ We hypothesized that a good dietary quality in mothers with overweight or obesity during pregnancy is associated with lower weight and adiposity in their children, while GDM may exert opposite effects on their children's adiposity. We aimed to investigate the extent to which the growth of children up to 24 months of age is influenced by (1) diet (nutrients and dietary quality) and (2) GDM during pregnancy.

## 2 METHODS

### 2.1 Ethics statement

The study was conducted according to the guidelines in the Declaration of Helsinki, and the Hospital District of Southwest Finland approved the study protocol. Each woman provided written informed consent before participation.

### 2.2 Participants and study design

**Figure 1:**
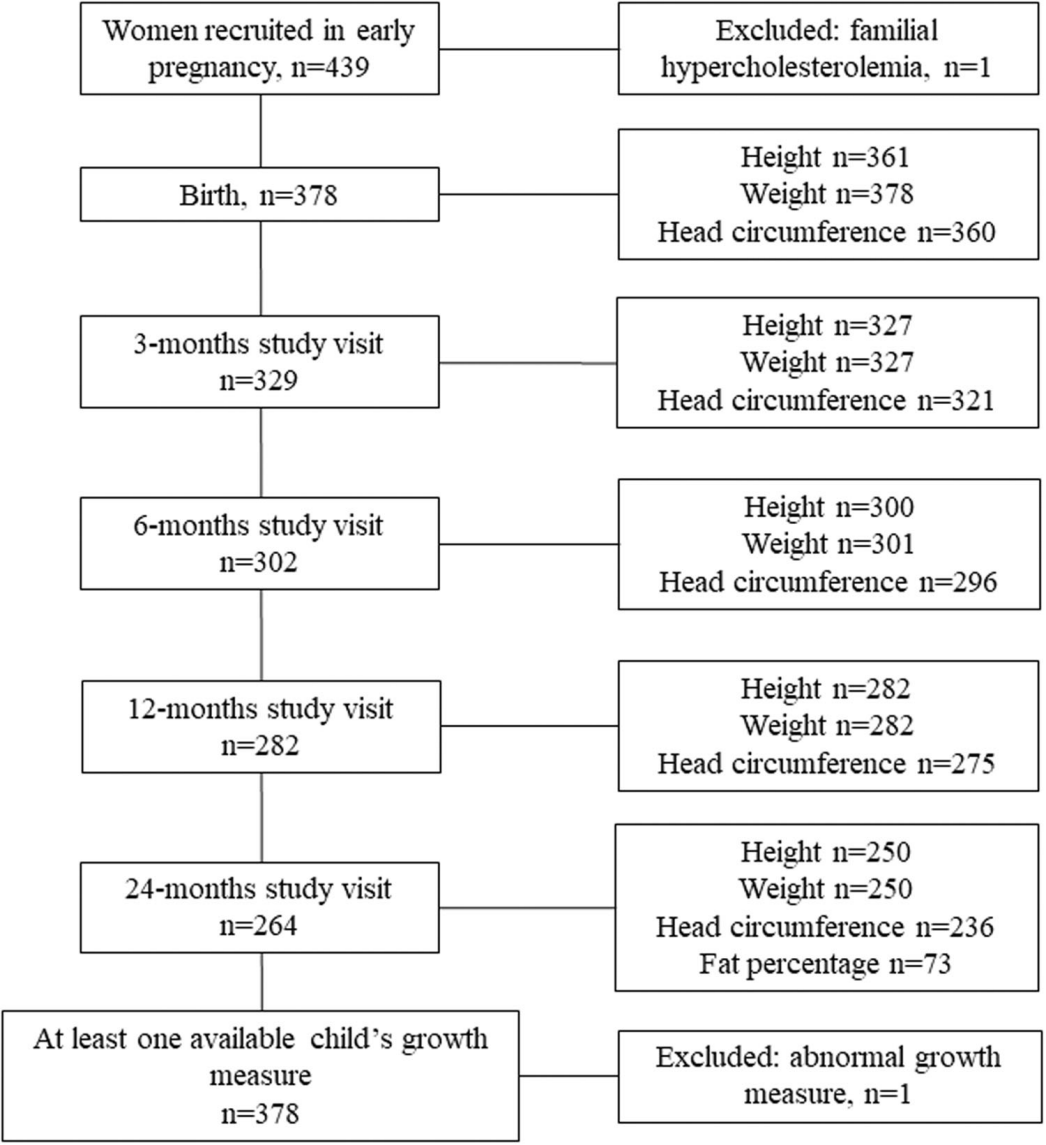
Flow chart of the present study. At least one available growth measure was available from 378 children during the study period (24 months).

Data for this study originated from a double‐blind, placebo‐controlled trial, described previously^16^ (ClinicalTrials.gov Identifier: NCT01922791). In total, 439 pregnant women (<18 gestational weeks [GW]) with overweight were recruited (2 October 2013 to 11 July 2017) in Southwest Finland. For the current study, we included children (*n* = 378) from whom we had at least one available growth measure during the study period (24 months) and their mothers (Figure 1).

Maternal diet was assessed in early (13.9 ± 2.1 GW) and late pregnancy (35.2 ± 0.98 GW). Children's growth data were gathered at delivery and at 3, 6, 12 and 24 months. The women's and children's background information was gathered through interviews and questionnaires during early pregnancy or from hospital medical records. The women were randomized into four intervention groups (fish oil+placebo, probiotics+placebo, fish oil+probiotics and placebo+placebo) at baseline, and the intervention lasted from early pregnancy until 6 months postpartum. The intervention has been described earlier.^16^

### 2.3 Dietary assessments

Dietary intake was recorded by 3‐day food diaries (2 weekdays, 1 weekend day). The mothers were instructed both orally and in writing on how to record their consumption of food and drinks. A picture portion booklet was used to estimate the correctness of portion sizes. Mean daily intakes of energy and energy‐yielding nutrients were calculated with a computerized software (AivoDiet 2.0.2.3; Aivo). The intakes of eicosapentaenoic acid (EPA, 0.22 g) and docosahexaenoic acid (DHA, 1.9 g) from fish oil‐intervention food supplements were added to the dietary intakes of the respective groups (total intake).

A validated Index of Diet Quality (IDQ) questionnaire^17^ was used to assess the overall quality of the diet. The questionnaire contains 18 questions concerning the frequency and amount of food groups during the preceding week: wholegrain bread, saturated/unsaturated fatty acids, dairy products, vegetables, fruits, berries, sugar‐containing drinks/sweets, <2 skipped meals. After scoring each question, the diet was of good (≥10/15) or poor (<10/15) quality as defined in the article describing the index's development.^17^

The dietary inflammatory index (DII®) and energy‐adjusted DII (E‐DII™) were used to assess diet‐associated inflammation.^18^, ^19^ Data were derived from the 3‐day food diaries. DII scores can range from strongly anti‐inflammatory (−8.87) to strongly pro‐inflammatory (+7.98). For this study a total of 28 foodparameters were available from the food diaries: energy, carbohydrates, protein, total fat, alcohol, fibre, cholesterol, SFA, monounsaturated fatty acid, PUFA, *n* − 3 and *n* − 6 fatty acids, trans‐fatty acids, iron, magnesium, zinc, selenium, folic acid, beta‐carotene, niacin, thiamine, riboflavin, vitamins B12, B6, A, C, D and E.

### 2.4 Maternal obesity and GDM

Pre‐pregnancy BMI (kg/m^2^) was calculated based on the women's self‐reported pre‐pregnancy weight and height measured during the first study visit with a wall stadiometer to the nearest 0.1 cm. Mothers were classified as having overweight or obesity. Body composition was measured using air displacement plethysmography (the Bod Pod System, software version 5.4.0, COSMED, Inc.) according to the manufacturer's instructions as described earlier^20^ and body fat percentage was calculated using the formulas devised by van Raaij et al.^21^ GDM was diagnosed with a 2‐h oral glucose tolerance test according to Finnish current care guidelines^22^ that are in accordance with American Diabetes Association 2007 guidelines. The test was offered in the maternal welfare clinics and was conducted in mid‐pregnancy (median: 25.9, interquartile range: 25.0–27.4 GW) or in early pregnancy (median: 14.7, interquartile range: 13.0–16.0 GW) for women at high risk.^22^

### 2.5 Children's anthropometrics and body composition

The children's weight, height, and head circumference were obtained from child welfare clinic records. Standard deviation (SD) scores for height‐for‐age, weight‐for‐age, head circumference‐for‐age, and BMI‐for‐age as well as weight‐for‐height% were calculated according to the Finnish growth charts.^23^, ^24^ Reference values for BMI‐for‐age SD‐score were available for children whose decimal age was ≥1.995 years (*n* = 149).^24^ For children born pre‐term (*n* = 22), appropriate growth references were used.^25^ Children's body composition was measured at 24 months using air displacement plethysmography (the Bod Pod System, software version 5.4.0, COSMED, Inc.) according to the manufacturer's instructions applying the paediatric option of the Bod Pod System. Children were measured wearing a tight cap, underwear or swimming trunks without a diaper, and they did not have restrictions regarding eating or drinking before the measurement. The density model devised by Fomon et al.^26^ was used to calculate the fat percentage.

### 2.6 Statistical analysis

The normality of data was checked visually from histograms. Normally distributed variables were summarized as mean ± SD, while medians (interquartile range) were used for variables that were not normally distributed. Independent samples *t* test and Mann‐Whitney *U* test were used in comparisons. Categorical variables were described as frequency (percentage), with chi‐squared or Fisher's exact test being used in comparisons. General linear models were used to investigate the associations between a dichotomized dietary quality, GDM status and growth variables after adjusting for potential confounders. As the study population consisted of women with overweight/obesity, their potential effects were also investigated. Pearson or Spearman partial correlation coefficients were used when investigating the correlations between maternal nutrient intakes, diet inflammatory potential, body composition and growth variables. *p* Values from the correlation analysis were corrected for multiple analyses by the Benjamini–Hochberg method (false discovery rate 0.05). The analyses were adjusted for birthweight (except for weight‐for‐age SD‐score and weight‐for‐height% at birth) as it is known to affect growth, and the analysis with weight‐for‐height% as an outcome for a child's age (3–24 months). In addition, confounders were selected based on group differences: (1) dietary quality: education level (early and late pregnancy), pre‐pregnancy smoking (early pregnancy) and maternal age (late pregnancy); (2) GDM‐status: education level, pre‐pregnancy BMI and gestational age; (3) obesity status: GDM‐status, gestational age. As gestational age correlated with birthweight, they were not included in the same model. The original trial intervention groups (fish oil and/or probiotics or placebo) were considered as confounders in general linear models. Fat percentage was natural log transformed for the GDM and IDQ (early pregnancy) analyses due to its skewed distribution. Statistical analyses were conducted using IBM SPSS Statistics version 27 for Windows (IBM Corp). *p* Values < 0.05 were considered statistically significant.

## 3 RESULTS

### 3.1 Study participants

Nearly half of the women (47.5%) had a good dietary quality (Table 1). These women had a higher education level, and they were less likely to smoke before and during pregnancy when compared to those with a poor dietary quality. Women with a good dietary quality breastfed longer compared to women with a poor dietary quality. Of the women, nearly 30% were diagnosed with GDM, and 40% were classified as having obesity. The women without GDM had a higher education level and a lower pre‐pregnancy BMI when compared to those with GDM (Table 1). No difference was seen in GDM diagnosis according to the women's dietary quality. The children's characteristics did not differ according to the maternal dietary quality or GDM‐status groups.

**Table 1:** Clinical characteristics of all mothers and their children were assessed according to the maternal dietary quality, defined as good or poor, in early pregnancy and GDM status.

Characteristics	All		Good dietary quality	Poor dietary quality			Non‐GDM	GDM	
Mother	*n* = 378	*n*	*n* = 178	*n* = 197	*p* d	*n*	*n* = 263	*n* = 107	*p* d
Age (years)^a^	30.6 ± 4.54	178/197	31.0 ± 4.38	30.1 ± 4.66	0.056	263/107	30.4 ± 4.45	31.2 ± 4.69	0.104
College or university education^b^	228 (61.5)	175/193	128 (73.1)	99 (51.3)	<0.001*	257/106	169 (65.8)	56 (52.8)	0.024*
Primiparity^b^	183 (48.4)	178/197	84 (47.2)	99 (50.3)	0.605	263/107	127 (48.3)	51 (47.7)	1.000
Smoked before pregnancy^b^	79 (21.2)	176/194	26 (14.8)	52 (26.8)	0.005*	257/106	59 (22.8)	16 (15.1)	0.117
Pre‐pregnancy BMI (kg/m^2^)^c^	28.7 (26.5–32.0)	178/197	28.7 (26.5–31.9)	28.7 (26.5–32.3)	0.997	263/107	28.3 (26.3–31.2)	30.1 (27.5–33.8)	<0.001*
With overweight^b^	228 (60.3)	178/197	106 (59.6)	119 (60.4)	0.916	263/107	173 (65.8)	52 (48.6)	0.003*
With obesity^b^	150 (39.7)		72 (40.4)	78 (39.6)			90 (34.2)	55 (51.4)	
GDM diagnosis in current pregnancy^b^	107 (28.9)	174/193	42 (24.1)	64 (32.2)	0.065	–	–	–	
Smoked during pregnancy^b^	19 (5.1)	174/194	4 (2.3)	15 (7.7)	0.019*	259/106	13 (5.1)	4 (3.8)	0.787
Unassisted vaginal delivery^b^	277 (73.3)	178/197	129 (72.5)	145 (73.6)	0.817	263/107	197 (74.9)	75 (70.1)	0.364
Gestational weeks at delivery^c^	39.6 (39.0–40.6)	178/197	40.0 (39.0–40.6)	39.9 (39.0–40.6)	0.982	263/107	40.1 (39.1–40.7)	39.4 (38.4–40.4)	<0.001*
Child									
Sex, girl^b^	193 (51.1)	178/197	99 (55.6)	92 (46.7)	0.098	263/107	136 (51.7)	54 (50.5)	0.909
Born preterm^b^	22 (5.8)	178/197	8 (4.1)	14 (7.9)	0.129	263/107	15 (5.7)	7 (6.5)	0.809
SGA^b^	13 (3.4)	178/197	7 (3.9)	6 (3.0)	0.779	263/107	7 (2.7)	6 (5.6)	0.211
LGA^b^	16 (4.2)	178/197	5 (2.8)	11 (5.6)	0.210	263/107	11 (4.2)	5 (4.7)	0.784
Breastfeeding (months)^a^	11.0 ± 6.71	125/147	11.8 ± 6.39	10.2 ± 6.88	0.043*	189/80	10.8 ± 6.37	11.6 ± 7.52	0.411

*Note*: Data are presented as ^a^mean ± SD, ^b^frequency (%) or ^c^median (interquartile range).

Abbreviations: BMI, body mass index; GDM, gestational diabetes mellitus; LGA, large for gestational age; SD, standard deviation; SGA, small for gestational age.

^d^ Independent samples *t* test for normally distributed variables, Mann–Whitney *U* test for non‐normally distributed variables, chi‐squared test or Fisher test for categorical variables.

* Significant value (*p* < 0.05).

### 3.2 Maternal diet during pregnancy and child's growth

Generally, the mean growth of children was within the normal reference range (Table 2). Most of the children were classified as having normal weight (81.6%) and the rest as having overweight or obesity. A good dietary quality in early pregnancy associated with an approximately 0.3 SD higher mean height‐for‐age SD‐score at each timepoint, and similarly a higher mean head circumference‐for‐age SD‐score at 12 and 24 months (Table 2). A good dietary quality in late pregnancy associated with a lower fat mass in 24‐month‐old children but not with other growth markers from birth until 24 months (Table S1).

**Table 2:** Association between the maternal dietary quality, defined as good or poor, in early pregnancy and the child's growth during the first 24 months of age.

			Good dietary quality	Poor dietary quality	Adjusted mean difference (95% CI)	
Growth variables	All (mean ± SD)	*n*	Adjusted mean (SE)	Adjusted mean (SE)	Good–Poor dietary quality	Adjusted *p* a
Birth						
Height‐for‐age SD‐score	0.04 ± 1.00	168/190	0.19 (0.08)	−0.10 (0.06)	0.28 (0.10–0.46)	0.002*
Weight‐for‐height%	2.14 ± 9.37	154/182	1.19 (0.92)	2.91 (0.77)	−1.72 (−3.84 to 0.40)	0.112
Weight‐for‐age SD‐score	0.21 ± 1.06	164/189	0.19 (0.10)	0.18 (0.09)	0.01 (−0.23 to 0.25)	0.935
Head circumference‐for‐age SD‐score	0.21 ± 1.03	168/189	0.28 (0.08)	0.10 (0.07)	0.17 (−0.01 to 0.36)	0.069
3 months						
Height‐for‐age SD‐score	−0.20 ± 1.11	159/165	−0.04 (0.09)	−0.33 (0.08)	0.28 (0.07–0.50)	0.008*
Weight‐for‐height%	3.14 ± 8.40	159/165	2.93 (0.85)	4.51 (0.76)	−1.58 (−3.50 to 0.34)	0.106
Weight‐for‐age SD‐score	−0.02 ± 0.97	159/165	0.08 (0.09)	0.00 (0.08)	0.08 (−0.12 to 0.28)	0.445
Head circumference‐for‐age SD‐score	−0.07 ± 1.10	154/164	0.12 (0.11)	−0.11 (0.09)	0.23 (−0.01 to 0.47)	0.056
6 months						
Height‐for‐age SD‐score	−0.26 ± 1.11	145/152	−0.01 (0.10)	−0.31 (0.09)	0.30 (0.07–0.54)	0.011*
Weight‐for‐height%	4.34 ± 8.47	144/152	4.19 (0.90)	5.36 (0.81)	−1.17 (−3.20 to 0.86)	0.258
Weight‐for‐age SD‐score	0.06 ± 0.95	144/152	0.19 (0.09)	0.12 (0.08)	0.08 (−0.14 to 0.29)	0.484
Head circumference‐for‐age SD‐score	−0.03 ± 1.08	141/151	0.18 (0.11)	−0.06 (0.10)	0.24 (−0.001 to 0.48)	0.051
12 months						
Height‐for‐age SD‐score	−0.19 ± 1.08	138/141	0.06 (0.11)	−0.22 (0.10)	0.28 (0.03–0.52)	0.025*
Weight‐for‐height%	2.76 ± 8.30	138/141	3.90 (0.88)	4.53 (0.80)	−0.63 (−2.62 to 1.37)	0.536
Weight‐for‐age SD‐score	0.01 ± 0.98	138/141	0.25 (0.10)	0.14 (0.09)	0.10 (−0.12 to 0.32)	0.371
Head circumference‐for‐age SD‐score	−0.10 ± 1.09	133/139	0.20 (0.11)	−0.18 (0.10)	0.38 (0.12–0.63)	0.004*
24 months						
Height‐for‐age SD‐score	−0.17 ± 1.06	124/124	0.04 (0.11)	−0.24 (0.10)	0.28 (0.02–0.54)	0.034*
Weight‐for‐height%	2.91 ± 8.52	124/124	3.89 (0.93)	4.18 (0.84)	−0.29 (−2.43 to 1.85)	0.789
Weight‐for‐age SD‐score	0.06 ± 0.99	124/124	0.25 (0.11)	0.12 (0.13)	0.13 (−0.11 to 0.37)	0.275
Head circumference‐for‐age SD‐score	−0.06 ± 1.06	114/120	0.20 (0.12)	−0.22 (0.10)	0.42 (0.15–0.68)	0.002*
BMI‐for‐age SD‐score	0.28 ± 1.05	70/79	0.37 (0.15)	0.44 (0.13)	−0.07 (−0.42 to 0.28)	0.690
Fat percentage	24.6 ± 8.87	35/38	23.5 (20.8–26.6)	22.5 (20.0–25.3)	1.05 (0.88–1.24)	0.604
Fat mass (kg)	3.30 ± 1.48	35/38	3.32 (0.24)	3.28 (0.23)	0.04 (−0.63 to 0.71)	0.899
Fat‐free mass (kg)	9.78 ± 1.01	35/38	9.79 (0.17)	9.80 (0.16)	−0.03 (−0.50 to 0.45)	0.907

*Note*: Data are presented as adjusted mean (SE) and adjusted mean difference (95% CI), except as adjusted geometric mean (95% CI) and proportional difference for adjusted geometric mean (95% CI) for fat percentage. Fat percentage is ln transformed in the analysis due to its skewed distribution.

Abbreviations: BMI, body mass index; CI, confidence interval; SD, standard deviation; SE, standard error.

^a^ General linear model, adjusted for maternal education level, pre‐pregnancy smoking status, child's birth weight (except for birth weight variables), child's age (weight‐for‐height%, 3–24 months) and intervention groups.

* Significant value (*p* < 0.05).

When investigating diet inflammatory potential, we found that a higher DII correlated negatively with children's height measures, while positive correlations were detected with children's weight measures (see details in Figure S2). We detected associations between dietary quality (early pregnancy 9.50 ± 2.08, late pregnancy 9.67 ± 2.04), a DII (early pregnancy −0.49 ± 1.78, late pregnancy −0.52 ± 1.74) and an E‐DII (early pregnancy −1.16 ± 1.61, late pregnancy, −1.10 ± 1.63); negative correlations were observed between IDQ and DII/E‐DII in early pregnancy (DII *r* = −0.38, *p* < 0.001 and E‐DII *r* = −0.41, *p* < 0.001) and late pregnancy (DII *r* = −0.29, *p* < 0.001, E‐DII *r* = −0.40, *p* < 0.001).

We inspected the mothers' diets in more detail by evaluating their nutrient intakes. In early and late pregnancy, positive correlations were seen between the intakes of total fat, SFA and children's weight or height measures, while negative correlations were detected between the intakes of PUFA, *n* − 6 fatty acids, *n* − 3 fatty acids, fibre and children's weight measures (see details in Figure S3).

### 3.3 Maternal GDM, obesity status and child's growth

GDM associated with an approximately 0.2 SD lower mean head circumference‐for‐age SD‐score of children at birth and 6 months of age, but not with other growth markers (Table 3). We also investigated whether maternal obesity influences the growth markers, but did not find any associations (Table S4). Finally, the effects of GDM and obesity together on children's growth were evaluated, but no associations were detected (Table S5). When investigating maternal adiposity in more detail, we detected positive correlations between maternal fat mass and children's height and head circumference (Figure S6).

**Table 3:** Association between the maternal GDM status and the child's growth during the first 24 months of age.

		Without GDM	With GDM	Adjusted mean difference (95% CI)	
Growth variables	*n*	Adjusted mean (SE)	Adjusted mean (SE)	GDM–non‐GDM	Adjusted *p* a
Birth					
Height‐for‐age SD‐score	249/104	0.08 (0.05)	0.01 (0.08)	−0.07 (−0.26 to 0.13)	0.493
Weight‐for‐height%	243/97	2.59 (0.66)	1.00 (1.00)	−1.59 (−4.01 to 0.82)	0.195
Weight‐for‐age SD‐score	248/100	0.22 (0.07)	0.18 (0.10)	−0.04 (−0.28 to 0.21)	0.776
Head circumference‐for‐age SD‐score	248/104	0.28 (0.06)	0.06 (0.09)	−0.22 (−0.43 to −0.02)	0.030*
3 months					
Height‐for‐age SD‐score	228/93	−0.20 (0.07)	−0.27 (0.10)	−0.07 (−0.30 to 0.17)	0.562
Weight‐for‐height%	228/93	3.34 (0.59)	2.95 (0.89)	−0.39 (−2.49 to 1.72)	0.717
Weight‐for‐age SD‐score	228/93	0.01 (0.06)	−0.10 (0.09)	−0.11 (−0.33 to 0.12)	0.355
Head circumference‐for‐age SD‐score	226/89	−0.01 (0.07)	−0.24 (0.11)	−0.23 (−0.49 to 0.03)	0.087
6 months					
Height‐for‐age SD‐score	211/83	−0.29 (0.07)	−0.25 (0.11)	0.03 (−0.23 to 0.30)	0.811
Weight‐for‐height%	211/83	4.58 (0.62)	2.75 (0.95)	−1.83 (−4.06 to 0.41)	0.109
Weight‐for‐age SD‐score	211/82	0.07 (0.07)	−0.06 (0.10)	−0.13 (−0.37 to 0.11)	0.297
Head circumference‐for‐age SD‐score	207/82	0.03 (0.08)	−0.24 (0.12)	−0.28 (−0.55 to −0.004)	0.047*
12 months					
Height‐for‐age SD‐score	196/80	−0.22 (0.08)	−0.22 (0.12)	−0.004 (−0.28 to 0.27)	0.978
Weight‐for‐height%	196/80	3.14 (0.62)	1.27 (0.93)	−1.87 (−4.08 to 0.33)	0.096
Weight‐for‐age SD‐score	196/80	0.02 (0.07)	−0.14 (0.11)	−0.16 (−0.41 to 0.09)	0.218
Head circumference‐for‐age SD‐score	192/78	−0.07 (0.08)	−0.31 (0.12)	−0.24 (−0.53 to 0.05)	0.100
24 months					
Height‐for‐age SD‐score	178/66	−0.17 (0.08)	−0.24 (0.13)	−0.07 (−0.37 to 0.23)	0.661
Weight‐for‐height%	178/66	2.92 (0.66)	2.06 (1.01)	−0.86 (−3.22 to 1.50)	0.472
Weight‐for‐age SD‐score	178/66	0.05 (0.08)	−0.04 (0.12)	−0.09 (−0.36 to 0.18)	0.520
Head circumference‐for‐ age SD‐score	166/64	−0.02 (0.09)	−0.28 (0.13)	−0.26 (−0.56 to 0.04)	0.091
BMI‐for‐age SD‐score	104/40	0.18 (0.11)	0.35 (0.17)	0.17 (−0.22 to 0.56)	0.384
Fat percentage	49/21	22.4 (20.1–24.9)	23.2 (19.6–27.3)	1.04 (0.85–1.26)	0.713
Fat mass (kg)	49/21	3.25 (0.20)	3.49 (0.32)	0.24 (−0.53 to 1.01)	0.535
Fat‐free mass (kg)	49/21	9.84 (0.15)	9.52 (0.23)	−0.33 (−0.88 to 0.23)	0.244

*Note*: Data are presented as adjusted mean (SE) and adjusted mean difference (95% CI), except as adjusted geometric mean (95% CI) and proportional difference for adjusted geometric mean (95% CI) for fat percentage. Fat percentage is ln transformed in the analysis due to its skewed distribution.

Abbreviations: BMI, body mass index; CI, confidence interval; SD, standard deviation; SE, standard error.

^a^ General linear model, adjusted for the maternal pre‐pregnancy BMI, education level, child's birth weight (except for birth weight variables) or gestational weeks at delivery (weight‐for‐age SD‐score and weight‐for‐height% at birth), child's age (weight‐for‐height%, 3–24 months) and intervention groups.

* Significant value (*p* < 0.05).

## 4 DISCUSSION

In this study, we demonstrated that early‐life factors, such as maternal diet and GDM, influence childhood growth. Children of mothers consuming a good quality diet were taller and had a larger head circumference but a lower fat mass up to 24 months of age in comparison to those of mothers with a poor dietary quality. We also detected modest correlations between maternal nutrient intakes and children's growth. The inflammatory potential of the maternal diet could partly explain these associations. Although the mean growth of children was normal, we found that the presence of GDM might result in the child having a smaller head circumference.

Our findings suggest that a good dietary quality in early pregnancy is linked with better growth of children, that is, greater height and a larger head circumference over the first 24 months of life. Our finding is in line with previous trials reporting a positive association between a better maternal dietary quality in early pregnancy, defined by the alternate Mediterranean diet score and Dietary Approaches to Stop Hypertension (*n* = 1948)^10^ and the alternate Healthy Eating Index (*n* = 787)^27^ and children's height. Further, a higher consumption of seafood by the mother has been linked with a greater head circumference of children at birth (*n* = 62,099).^28^ In contrast to our study, these studies included both women with normal weight and overweight, and the assessment point was only at birth. We also found that 2‐year‐old children of mothers with a good dietary quality in late pregnancy had a lower fat mass when compared to those of mothers with a poor dietary quality. Support for our result arises from a previous study (*n* = 354), which found that a better dietary quality (the Healthy Eating Index) from pregnancy until 3 months postpartum associated with a lower body fat percentage in 6‐month‐old children.^9^ However, another study (*n* = 2695) did not find that data derived dietary patterns (‘vegetable‐fish‐oil’, ‘nuts‐soy‐high‐fibre cereals’ and ‘margarine‐snacks‐sugar’) in pregnancy exerted any influence on the fat percentage in 6‐year‐old children.^29^ These apparent differences could be due to the differing methods applied in the diet assessments as well as in the study populations. As adipose tissue has a key role in mediating the unfavourable effects of obesity, the putative impacts of the maternal diet on a child's adiposity should be further clarified.

Our finding that a good dietary quality during pregnancy has beneficial effects on children's growth could be explained by its nutritional content, that is, a higher consumption of vegetables, fruits, berries and fish. These foods contain many vitamins and minerals, for example, folate, vitamin D and iodine that are vital for the child's brain development and growth.^30^, ^31^, ^32^ In fact, in the evaluation of maternal nutrient intakes, we detected that especially higher intakes of anti‐inflammatory *n* − 3 PUFAs, namely EPA and DHA, as well as that of fibre associated with a better growth in the children, findings that have been reported previously.^12^, ^13^, ^33^ On the other hand, a poor diet composition, characterized by a higher intake of fat, especially SFA, increases the level of low‐grade inflammation in the body.^15^ Indeed, our findings together with published reports^14^, ^34^, ^35^, ^36^ suggest that a higher intake of SFA and/or a higher inflammatory potential in the maternal diet may lead to an unfavourable growth of children, that is, greater weight and reduced height. Thus, the diet‐induced inflammation may partly explain how a poor dietary quality of mother influences children's growth. This concept is further supported by our finding that IDQ and DII correlated negatively, indicating that an overall good dietary quality is less inflammatory.

One novel, and somewhat unexpected finding is that GDM during pregnancy led to a slightly smaller head circumference of children at birth and 6 months, although the mean growth of children was normal. As far as we are aware, previous investigators have not observed a similar association, although one recent study (*n* = 454) found relations between an impaired glucose tolerance, including GDM and type 2 diabetes, during pregnancy and a reduced size of an infant in early childhood, that is, lower height, weight, and BMI.^37^ Thus, clinical significance of our finding remains unclear, calling for further research on this topic. One underlying mechanism for our finding could be that GDM alters placental function so that the transport of some nutrients, such as DHA, may be reduced.^38^ In fact, it has been shown that the transport of DHA, a compound known to be important for brain development, is reduced in pregnancies affected by GDM.^39^ This is putatively due to a lower level of the DHA transporter in blood of GDM mothers, which was also found to associate with a smaller head circumference of children.^39^ It is well known that GDM increases the risk for macrosomia and weight‐problems in childhood,^3^, ^4^, ^6^ mainly due to the elevated maternal glucose level during pregnancy. However, in our study, these associations were not detected. In Finland, GDM is routinely screened, and all women with GDM receive lifestyle guidance, combined with medical treatment if needed, in the maternal welfare clinics.^22^ Thus, all women with GDM in our study received lifestyle or medical treatment that could, to some extent, explain why GDM did not lead to higher growth measures of the children. We did not find associations between maternal overweight/obesity and children's growth. However, when using a more accurate method to assess mothers' adiposity, that is, body composition measurement, we detected positive associations between maternal fat mass and height and head circumference measures of children. To our knowledge, no previous studies have investigated the long‐term effects of maternal body composition during pregnancy on children's growth, thus this novel finding should be confirmed in future studies.

Our findings regarding the associations between GDM, dietary quality and children's head circumference could provide clarification on how these early‐life factors affect childhood neurodevelopment. Emerging evidence from us and others has shown that GDM has untoward effects on children's neurodevelopmental performance while a good dietary quality has the opposite effect.^40^, ^41^, ^42^ The association between a smaller head circumference and a poorer neurodevelopment of children has been shown in a recent study, although that population consisted of children born pre‐term.^43^ Besides, GDM has been linked with anatomical brain differences in 6‐year‐old children born to mother with overweight.^44^ Our findings indicate that it could be possible to benefit children's growth and thus neurodevelopment by improving mother's diet especially in early pregnancy. A healthy diet may exert various health benefits for pregnant women, such as preventing the onset of GDM.^45^ In addition, it may also benefit children's growth and development. According to previous reports the dietary intake of Finnish pregnant women, especially those at risk for GDM, on average do not meet the Finnish diet recommendations in all respects, in particular that of SFA, *n* – 3 PUFA, sucrose, and fibre,^45^, ^46^, ^47^, ^48^ which demonstrates that there is a need to pay more attention to dietary counselling of these women.

One strength of our study is its prospective design, which enabled us to evaluate the associations between early life factors and children's growth from birth to 24 months of age. In addition, as the data were obtained in a clinical trial setting, we were able to collect and take into account many possible confounding factors. We had growth data available from five time points and body composition data at 24 months. Also, we used a previously validated dietary assessment tool, IDQ and calculated DII to evaluate the diet's inflammatory potential. The intervention did not influence the primary outcomes of the main trial (maternal glucose metabolism)^16^; nevertheless, we considered it a potential confounder in the analysis as it may affect the child's growth through other biological pathways. Despite its strengths, the study has limitations. First, our study population consisted of women with overweight/obesity, thus we did not have women with normal weight as a control group. However, overweight is common in Finland and globally,^1^, ^49^ and these women represent typical clients of maternal welfare clinics. Further limitation is drop‐out over the course of the study. We found that maternal dietary quality associated with a child's fat mass but not with fat percentage; thus, further studies are needed to verify this finding. It is of note that postnatal factors, such as mothers' and children's lifestyle and diet, including a child's feeding, influence the long‐term growth. Thus, a further limitation is that we did not adjust the analysis for breastfeeding, as the data were not available from all the mothers.

## 5 CONCLUSION

These are the first results suggesting that GDM may lead to a slightly smaller head circumference in children, although their growth was within the normal reference range. In contrast, consuming a good quality diet during pregnancy may support the optimal growth of children at least up to 24 months of age. Considering that GDM is a growing health problem due to the increased prevalence of obesity, in the maternal welfare clinics, more attention should be paid to improve the dietary quality of high‐risk women, that is, those with overweight/obesity, already in early pregnancy. Dietary counselling may represent a cost‐effective way not only to yield health benefits for the women, but also to support their children's growth and development.

## CONFLICT OF INTEREST STATEMENT

Dr. James R. Hébert discloses that he owns controlling interest in Connecting Health Innovations LLC (CHI), a company that has licensed the right to his invention of the dietary inflammatory index (DII®) from the University of South Carolina to develop computer and smartphone applications for patient counselling and dietary intervention in clinical settings. CHI owns exclusive rights to the energy‐adjusted DII (E‐DII™). The subject matter of this paper has no direct bearing on that work, nor has any CHI‐related activity exerted any influence on this project. The remaining authors declare no conflicts of interest.

## Supporting information

Supporting information.

Supporting information.

Supporting information.

Supporting information.

Supporting information.

Supporting information.
