# Supplementary material for: Maternal diet and gestational diabetes mellitus modestly influence children's growth during their first 24 months
Source: J Pediatr Gastroenterol Nutr. 2025 Jun 9;81(2):355–66. doi: 10.1002/jpn3.70098 (PMC12314585; doi:10.1002/jpn3.70098)
Supplement: Supplementary file 1 — Supporting information. [file JPN3-81-355-s006.docx]

Supplemental digital content 1

Maternal diet and gestational diabetes mellitus modestly influence children’s growth during their first 24-months

Journal of Pediatric Gastroenterology and Nutrition

Table, Association between the maternal dietary quality, defined as good or poor, in late pregnancy and the child’s growth during the first 24 months of age

| Growth variables | n | Good dietary quality  Adjusted mean (SE) | Poor dietary quality  Adjusted mean (SE) | Adjusted mean difference (95% CI)  Good–Poor dietary quality | Adjusted p ^†^ |
| --- | --- | --- | --- | --- | --- |
| Birth |  |  |  |  |  |
| Height-for-age SD-score | 189/162 | 0.07 (0.06) | 0.02 (0.06) | 0.05 (-0.12; 0.23) | 0.534 |
| Weight-for-height% | 179/156 | 2.20 (0.75) | 1.99 (0.78) | 0.21 (-1.90; 2.32) | 0.845 |
| Weight-for-age SD-score | 190/162 | 0.22 (0.08) | 0.18 (0.09) | 0.04 (-0.20; 0.28) | 0.739 |
| Head circumference-for-age SD-score | 189/161 | 0.23 (0.07) | 0.18 (0.07) | 0.05 (-0.14; 0.23) | 0.614 |
| 3 months |  |  |  |  |  |
| Height-for-age SD-score | 176/142 | -0.14 (0.07) | -0.34 (0.08) | 0.20 (-0.01; 0.41) | 0.060 |
| Weight-for-height% | 176/142 | 3.45 (0.68) | 3.31 (0.73) | 0.15 (-1.79; 2.08) | 0.883 |
| Weight-for-age SD-score | 176/142 | 0.06 (0.07) | -0.11 (0.08) | 0.17 (-0.03; 0.37) | 0.100 |
| Head circumference-for-age SD-score | 172/140 | 0.00 (0.09) | -0.20 (0.09) | 0.20 (-0.04, 0.44) | 0.110 |
| 6 months |  |  |  |  |  |
| Height-for-age SD-score | 163/128 | -0.21 (0.09) | -0.37 (0.09) | 0.17 (-0.07; 0.41) | 0.171 |
| Weight-for-height% | 163/128 | 4.22 (0.73) | 4.51 (0.77) | -0.29 (-2.34; 1.77) | 0.784 |
| Weight-for-age SD-score | 162/128 | 0.08 (0.08) | 0.01 (0.08) | 0.07 (-0.15; 0.29) | 0.542 |
| Head circumference-for-age SD-score | 160/126 | 0.010 (0.09) | -0.14 (0.09) | 0.15 (-0.10; 0.39) | 0.244 |
| 12 months |  |  |  |  |  |
| Height-for-age SD-score | 157/116 | -0.19 (0.09) | -0.23 (0.10) | 0.04 (-0.21; 0.29) | 0.754 |
| Weight-for-height% | 157/116 | 2.62 (0.72) | 3.09 (0.80) | -0.47 (-2.56; 1.61) | 0.654 |
| Weight-for-age SD-score | 157/116 | -0.002 (0.08) | 0.01 (0.09) | -0.01 (-0.24; 0.23) | 0.940 |
| Head circumference-for-age SD-score | 153/113 | -0.05 (0.09) | -0.23 (0.10) | 0.18 (-0.09; 0.45) | 0.180 |
| 24 months |  |  |  |  |  |
| Height-for-age SD-score | 142/102 | -0.19 (0.09) | -0.24 (0.10) | 0.04 (-0.22; 0.31) | 0.749 |
| Weight-for-height% | 142/102 | 2.32 (0.77) | 3.86 (0.84) | -1.54 (-3.73; 0.66) | 0.170 |
| Weight-for-age SD-score | 142/102 | -0.01 (0.09) | 0.09 (0.10) | -0.10 (-0.35; 0.15) | 0.443 |
| Head circumference-for age SD-score | 132/98 | -0.07 (0.10) | -0.17 (0.11) | 0.09 (-0.18; 0.18) | 0.501 |
| BMI-for-age SD-score | 81/67 | 0.16 (0.13) | 0.43 (0.13) | -0.28 (-0.63; 0.08) | 0.125 |
| Fat percentage | 38/33 | 23.2 (1.38) | 26.4 (1.48) | -3.15 (-7.26; 0.95) | 0.130 |
| Fat mass (kg) | 38/33 | 3.00 (0.23) | 3.70 (0.25) | -0.69 (-1.35; -0.10) | 0.047 |
| Fat free mass (kg) | 38/33 | 9.76 (0.17) | 9.82 (0.18) | -0.05 (-0.55; 0.44) | 0.830 |

Data are presented as adjusted mean (SE), adjusted mean difference (95% CI).

**^†^** General linear model, adjusted for the maternal education level and age due to group differences (data not shown), child’s birth weight (except for birth weight variables), child’s age (weight-for-height%, 3-24 months), and intervention groups.

CI=confidence interval, SD=standard deviation score, SE=standard error.
