# Supplementary material for: Maternal diet and gestational diabetes mellitus modestly influence children's growth during their first 24 months
Source: J Pediatr Gastroenterol Nutr. 2025 Jun 9;81(2):355–66. doi: 10.1002/jpn3.70098 (PMC12314585; doi:10.1002/jpn3.70098)
Supplement: Supplementary file 2 — Supporting information. [file JPN3-81-355-s001.docx]

Supplemental digital content 2

Maternal diet and gestational diabetes mellitus modestly influence children’s growth during their first 24-months

Journal of Pediatric Gastroenterology and Nutrition

| -0.2 | -0.15 | -0.1 | -0.05 | 0 | 0.05 | 0.1 | 0.15 | 0.2 | 0.25 |
| --- | --- | --- | --- | --- | --- | --- | --- | --- | --- |
|  |  |  |  |  |  |  |  |  |  |

|  | **Early pregnancy** | | **Late pregnancy** | |
| --- | --- | --- | --- | --- |
|  | DII | E-DII | DII | E-DII |
| **Birth** |  |  |  |  |
| Height SD-score | **† |  | **† |  |
| Weight-for-height % |  |  | * | * |
| Weight-for-age SD-score |  |  |  |  |
| Head circumference-for-age SD-score |  |  |  |  |
| **Three months** |  |  |  |  |
| Height SD-score | **† |  | **† |  |
| Weight-for-height % |  |  |  |  |
| Weight-for-age SD-score |  |  |  |  |
| Head circumference-for-age SD-score |  |  |  |  |
| **Six months** |  |  |  |  |
| Height SD-score | **† |  | **† |  |
| Weight-for-height % |  |  | * |  |
| Weight-for-age SD-score |  |  |  |  |
| Head circumference-for-age SD-score |  |  |  |  |
| **12 months** |  |  |  |  |
| Height SD-score | **† | **† | **† |  |
| Weight-for-height % | *† |  | *† |  |
| Weight-for-age SD-score |  |  |  |  |
| Head circumference-for-age SD-score |  |  |  |  |
| **24 months** |  |  |  |  |
| Height SD-score | **† |  | **† |  |
| Weight-for-height % | **† |  | **† |  |
| Weight-for-age SD-score |  |  |  |  |
| Head circumference-for-age SD-score | * |  |  |  |
| BMI-for-age SD-score | *† |  | **† |  |
| Fat percentage |  |  |  |  |
| Fat mass (kg) |  |  |  |  |
| Fat free mass (kg) |  |  |  |  |

Figure

Heatmap demonstrating the correlations between maternal diet inflammatory potential (DII and E-DII) in early and late pregnancy and the child’s growth markers during the first 24 months of age. The intensity of the colours represents the degree of Pearson’s partial correlation coefficient, red colour indicating a positive correlation and blue indicating a negative correlation (not corrected for multiple testing, *p<0.05, **p<0.01). Adjusted for birth weight (except for birth weight variables), and for the child’s age (weight-for-height%, 3-24 months). Corrected for multiple testing by the Benjamini-Hochberg procedure (†corrected p<0.05).

DII=Diet inflammatory index, E-DII=Energy-adjusted diet inflammatory index, SD-score=Standard deviation score
