# Supplementary material for: Maternal diet and gestational diabetes mellitus modestly influence children's growth during their first 24 months
Source: J Pediatr Gastroenterol Nutr. 2025 Jun 9;81(2):355–66. doi: 10.1002/jpn3.70098 (PMC12314585; doi:10.1002/jpn3.70098)
Supplement: Supplementary file 4 — Supporting information. [file JPN3-81-355-s004.docx]

Supplemental digital content 4

Maternal diet and gestational diabetes mellitus modestly influence children’s growth during their first 24-months

Journal of Pediatric Gastroenterology and Nutrition

Table, Association between the maternal obesity status, based on pre-pregnancy BMI, and the child’s growth during the first 24 months of age

| Growth variables | n | With overweight  Adjusted mean (SE) | With obesity  Adjusted mean (SE) | Adjusted mean difference (95% CI)  Obesity–Overweight | Adjusted p ^†^ |
| --- | --- | --- | --- | --- | --- |
| Birth |  |  |  |  |  |
| Height-for-age SD-score | 218/143 | 0.07 (0.06) | -0.01 (0.07) | -0.08 (-0.25; 0.10) | 0.381 |
| Weight-for-height% | 205/134 | 1.44 (0.74) | 2.43 (0.85) | 0.99 (-1.16; 3.13) | 0.367 |
| Weight-for-age SD-score | 215/141 | 0.18 (0.08) | 0.26 (0.09) | 0.09 (-0.13; 0.31) | 0.433 |
| Head circumference-for-age SD-score | 218/142 | 0.16 (0.06) | 0.21 (0.07) | 0.06 (-0.13; 0.24) | 0.546 |
| 3 months |  |  |  |  |  |
| Height-for-age SD-score | 198/129 | -0.19 (0.07) | -0.27 (0.09) | -0.09 (-0.30; 0.13) | 0.434 |
| Weight-for-height% | 198/129 | 3.11 (0.66) | 2.89 (0.76) | -0.22 (-2.13; 1.69) | 0.820 |
| Weight-for-age SD-score | 198/129 | -0.02 (0.07) | -0.08 (0.08) | -0.06 (-0.26; 0.14) | 0.571 |
| Head circumference-for-age SD-score | 195/126 | -0.13 (0.08) | -0.06 (0.10) | 0.07 (-0.17; 0.30) | 0.581 |
| 6 months |  |  |  |  |  |
| Height-for-age SD-score | 183/117 | -0.25 (0.08) | -0.26 (0.10) | -0.02 (-0.26; 0.23) | 0.905 |
| Weight-for-height% | 184/116 | 3.68 (0.70) | 3.85 (0.81) | 0.17 (-1.86; 2.20) | 0.871 |
| Weight-for-age SD-score | 183/116 | 0.02 (0.08) | 0.03 (0.09) | 0.02 (-0.20; 0.24) | 0.886 |
| Head circumference-for-age SD-score | 180/115 | -0.09 (0.09) | -0.05 (0.10) | 0.04 (-0.21; 0.28) | 0.774 |
| 12 months |  |  |  |  |  |
| Height-for-age SD-score | 173/109 | -0.24 (0.09) | -0.13 (0.10) | 0.11 (-0.15; 0.36) | 0.400 |
| Weight-for-height% | 173/109 | 2.34 (0.69) | 1.97 (0.81) | -0.37 (-2.41; 1.68) | 0.725 |
| Weight-for-age SD-score | 173/109 | -0.04 (0.08) | -0.05 (0.09) | -0.01 (-0.24; 0.22) | 0.938 |
| Head circumference-for-age SD-score | 171/104 | -0.15 (0.09) | -0.15 (0.11) | 0.002 (-0.27; 0.27) | 0.991 |
| 24 months |  |  |  |  |  |
| Height-for-age SD-score | 155/95 | -0.23 (0.09) | -0.13 (0.11) | 0.09 (-0.18; 0.37) | 0.496 |
| Weight-for-height% | 155/95 | 1.84 (0.73) | 3.60 (0.87) | 1.77 (-0.37; 3.90) | 0.105 |
| Weight-for-age SD-score | 155/95 | -0.05 (0.08) | 0.12 (0.10) | 0.17 (-0.08; 0.41) | 0.187 |
| Head circumference-for age SD-score | 144/92 | -0.15 (0.09) | -0.05 (0.11) | 0.10 (-0.18; 0.37) | 0.483 |
| BMI-for-age SD-score | 90/59 | 0.15 (0.12) | 0.45 (0.14) | 0.30 (-0.07; 0.66) | 0.108 |
| Fat percentage | 44/26 | 24.4 (1.32) | 25.6 (1.75) | 1.23 (-3.27; 5.73) | 0.588 |
| Fat mass (kg) | 44/26 | 3.20 (0.22) | 3.53 (0.29) | 0.33 (-0.42; -1.07) | 0.383 |
| Fat free mass (kg) | 44/26 | 9.71 (0.16) | 9.80 (0.21) | 0.08 (-0.45; 0.61) | 0.756 |

Data are presented as adjusted mean (SE), adjusted mean difference (95% CI).

**^†^** General linear model, adjusted for the maternal GDM status due to a group difference (data not shown), child’s birth (except for birth weight variables) or gestational weeks at delivery (weight-for-age SD-score and weight-for-height% at birth), child’s age (weight-for-height%, 3-24 months), and intervention groups.

CI=confidence interval, SD=standard deviation score, SE=standard error.
