# Supplementary material for: Maternal diet and gestational diabetes mellitus modestly influence children's growth during their first 24 months
Source: J Pediatr Gastroenterol Nutr. 2025 Jun 9;81(2):355–66. doi: 10.1002/jpn3.70098 (PMC12314585; doi:10.1002/jpn3.70098)
Supplement: Supplementary file 5 — Supporting information. [file JPN3-81-355-s005.docx]

Supplemental digital content 5

Maternal diet and gestational diabetes mellitus modestly influence children’s growth during their first 24-months

Journal of Pediatric Gastroenterology and Nutrition

Table, Association between the maternal combined gestational diabetes mellitus (GDM) and obesity, based on pre-pregnancy BMI, status and the child’s growth during the first 24 months of age

| Growth variables | n | With overweight, without GDM  Adjusted mean (SE) | With obesity, without GDM  Adjusted mean (SE) | With overweight and GDM  Adjusted mean (SE) | With obesity and GDM  Adjusted mean (SE) | p ^†^ |
| --- | --- | --- | --- | --- | --- | --- |
| Birth |  |  |  |  |  |  |
| Height-for-age SD-score | 164/51/85/53 | 0.11 (0.07) | 0.03 (0.11) | 0.02 (0.09) | -0.04 (0.11) | 0.665 |
| Weight-for-height% | 155/47/79/50 | 1.79 (0.79) | 1.57 (1.39) | 3.87 (1.12) | 0.99 (1.41) | 0.323 |
| Weight-for-age SD-score | 164/48/84/52 | 0.18 (0.08) | 0.14 (0.14) | 0.27 (0.12) | 0.27 (0.14) | 0.823 |
| Head circumference-for-age SD-score | 164/51/84/53 | 0.23 (0.07) | 0.12 (0.12) | 0.34 (0.10) | 0.07 (0.12) | 0.286 |
| 3 months |  |  |  |  |  |  |
| Height-for-age SD-score | 149/47/79/46 | -0.17 (0.08) | -0.23 (0.14) | -0.25 (0.11) | -0.35 (0.14) | 0.702 |
| Weight-for-height% | 149/47/79/46 | 3.31 (0.72) | 3.31 (1.23) | 3.31 (0.97) | 2.71 (1.25) | 0.977 |
| Weight-for-age SD-score | 149/47/79/46 | 0.02 (0.08) | -0.04 (0.13) | -0.01 (0.10) | -0.17 (0.13) | 0.676 |
| Head circumference-for-age SD-score | 148/45/78/44 | -0.08 (0.09) | -0.12 (0.16) | 0.09 (0.12) | -0.30 (0.16) | 0.250 |
| 6 months |  |  |  |  |  |  |
| Height-for-age SD-score | 141/40/70/43 | -0.28 (0.09) | -0.27 (0.16) | -0.30 (0.12) | -0.25 (0.15) | 0.996 |
| Weight-for-height% | 141/41/70/42 | 4.30 (0.75) | 3.38 (1.33) | 5.00 (1.02) | 2.38 (1.31) | 0.414 |
| Weight-for-age SD-score | 141/40/70/42 | 0.04 (0.08) | -0.02 (0.14) | 0.10 (0.11) | -0.09 (0.14) | 0.726 |
| Head circumference-for-age SD-score | 138/40/69/42 | -0.003 (0.09) | -0.20 (0.16) | 0.07 (0.13) | -0.23 (0.16) | 0.341 |
| 12 months |  |  |  |  |  |  |
| Height-for-age SD-score | 132/39/64/41 | -0.27 (0.09) | -0.23 (0.16) | -0.13 (0.13) | -0.18 (0.16) | 0.826 |
| Weight-for-height% | 132/39/64/41 | 3.30 (0.75) | 1.34 (1.31) | 2.77 (1.04) | 1.33 (1.28) | 0.416 |
| Weight-for-age SD-score | 132/39/64/41 | 0.02 (0.09) | -0.12 (0.15) | 0.02 (0.12) | -0.13 (0.15) | 0.710 |
| Head circumference-for-age SD-score | 130/39/62/39 | -0.11 (0.10) | -0.19 (0.17) | -0.02 (0.14) | -0.37 (0.17) | 0.416 |
| 24 months |  |  |  |  |  |  |
| Height-for-age SD-score | 118/35/60/31 | -0.21 (0.10) | -0.27 (0.17) | 0.12 (0.14) | -0.18 (0.19) | 0.904 |
| Weight-for-height% | 118/35/60/31 | 2.53 (0.80) | 0.66 (1.37) | 3.61 (1.06) | 4.12 (1.47) | 0.277 |
| Weight-for-age SD-score | 118/35/60/31 | 0.002 (0.09) | -0.14 (0.16) | 0.13 (0.12) | 0.11 (0.17) | 0.535 |
| Head circumference-for age SD-score | 108/34/58/30 | -0.07 (0.10) | -0.29 (0.17) | 0.05 (0.14) | -0.24 (0.19) | 0.339 |
| BMI-for-age SD-score | 71/18/33/22 | 0.06 (0.13) | 0.25 (0.24) | 0.38 (0.18) | 0.49 (0.22) | 0.281 |
| Fat percentage | 36/8/13/13 | 22.6 (19.9; 25.6) | 22.0 (17.1; 28.4) | 21.8 (17.8; 26.5) | 24.4 (19.6; 30.3) | 0.873 |
| Fat mass (kg) | 36/8/13/13 | 3.22 (0.23) | 3.09 (0.51) | 3.23 (0.39) | 3.84 (0.39) | 0.546 |
| Fat free mass (kg) | 36/8/13/13 | 9.82 (0.17) | 0.38 (0.37) | 9.92 (0.28) | 9.58 (0.28) | 0.591 |

Data are presented as adjusted mean (SE), except as adjusted geometric mean (95% CI) for fat percentage. Fat percentage is ln transformed in the analysis due to its skewed distribution.

**^†^** General linear model, adjusted for the maternal education level, birth weight (except for birth weight variables) or gestational weeks at delivery (weight-for-age SD-score and weight-for-height% at birth), child’s age (weight-for-height%, 3-24 months), and intervention groups.

CI=confidence interval, GDM=gestational diabetes mellitus, SD=standard deviation score, SE=standard error.
